# Analyses of the Complete Genome Sequence of the Strain *Bacillus pumilus* ZB201701 Isolated from Rhizosphere Soil of Maize under Drought and Salt Stress

**DOI:** 10.1264/jsme2.ME18096

**Published:** 2019-08-23

**Authors:** Zhongbao Zhang, Longfei Yin, Xianglong Li, Chun Zhang, Huawen Zou, Cai Liu, Zhongyi Wu

**Affiliations:** 1 Beijing Agro-Biotechnology Research Center, Beijing Academy of Agriculture and Forestry Sciences Beijing 100097 China; 2 College of Agriculture, Yangtze University, Hubei Collaborative Innovation Center for Grain Industry Jingzhou 434023, Hubei China; 3 College of Life Sciences, Capital Normal University Beijing 10048 China

**Keywords:** *Bacillus pumilus*, genome sequence, salinity and drought, rhizobacteria

## Abstract

*Bacillus pumilus* ZB201701 is a rhizobacterium with the potential to promote plant growth and tolerance to drought and salinity stress. We herein present the complete genome sequence of the Gram-positive bacterium *B. pumilus* ZB201701, which consists of a linear chromosome with 3,640,542 base pairs, 3,608 protein-coding sequences, 24 ribosomal RNAs, and 80 transfer RNAs. Genome analyses using bioinformatics revealed some of the putative gene clusters involved in defense mechanisms. In addition, activity analyses of the strain under salt and simulated drought stress suggested its potential tolerance to abiotic stress. Plant growth-promoting bacteria-based experiments indicated that the strain promotes the salt tolerance of maize. The complete genome of *B. pumilus* ZB201701 provides valuable insights into rhizobacteria-mediated salt and drought tolerance and rhizobacteria-based solutions for abiotic stress in agriculture.

Soil salinity and drought stress are increasingly serious examples of abiotic stress worldwide that limit plant growth and productivity (5, 10). Selected beneficial rhizobacteria may play an important role in promoting plant growth and tolerance to drought and salinity stress, thereby increasing crop yields (10, 14). Plant growth-promoting bacteria (PGPB) are free-living bacteria that form specific symbiotic relationships with plants or bacterial endophytes colonizing some or a part of the interior tissues of plants (1). PGPB are generally used as inoculants for biostimulation, biocontrol, and biofertilization. These bacteria may improve plant growth under different environmental conditions (24).

*Bacillus* species are important rhizobacteria that may improve plant growth and development via different mechanisms (19). *Bacillus pumilus* strains exhibit increased resistance to environmental biotic and abiotic stress and produce a wide range of industrial metabolites (7). They are found in a range of environments, from stratospheric air to deep-sea sediments, and from soil to living organisms (3, 17). Different strains of *B. pumilus* have been isolated from different rhizospheres and organisms and have been shown to exert various effects on hosts. For example, *B. pumilus* isolated from the rhizosphere of alder exhibits strong growth-promoting activity (9), while a strain isolated from the black tiger shrimp (*Penaeus monodon*) may be inhibitory against marine bacterial pathogens (12).

Maize, as an important source of food, feed, and industrial crops, is sensitive to drought and salt stress (22). In the present study, we aimed to select beneficial rhizobacteria that promote maize growth under drought and salt stress and analyze the complete genome sequences, activities of beneficial strains, and their ability to increase the salt resistance of maize.

## Materials and Methods

### Sample collection and bacteria isolation

The strain *B. pumilus* ZB201701 was isolated from the maize rhizosphere of drought-affected and saline soil in Bayan Nur of the Inner Mongolia Autonomous Region, China (40°13′–42°28′, E105°12′–109°53′). Soil was collected from the maize rhizosphere at a depth of 5–10 cm. Five points were selected according to the “S” form five-spot sampling method (18). One hundred grams of soil was collected at each point and combined into one composite soil sample. To isolate bacteria, soil samples were placed in paper bags and stored at 4°C for approximately 1 d. One hundred milliliters of sterile water and 10 g soil were then transferred into a Waring blender. The sample was homogenized for 1 min and the supernatant was collected.

Supernatants were centrifuged at 5,000×*g* for 10 min and then added to 0.5 mL sterile water. Dilutions of 10^−3^, 10^−4^, and 10^−5^ were placed on agar plates (5 g L^−1^ beef extract, 10 g L^−1^ peptone, 5 g L^−1^ NaCl, 100 g L^−1^ mannitol, 1 L distilled water, and 15 g L^−1^ agar; pH 7.2). After a 2-d incubation at 30°C, the biggest colony was transferred to 1.5-mL frozen pipes and stored at −80°C.

### Physiological characteristics and sequence similarity analysis

Physiological characteristics were identified based on Gram staining. A sequence similarity analysis was performed using 16S ribosomal RNA (rRNA) and two housekeeping genes (*recA* and *atpD*). A similarity search with the 16S rRNA gene nucleotide sequence (accession number MH368107) was conducted using EzBioCloud (https://www.ezbiocloud.net) (28) and average nucleotide identity (ANI) was calculated using JspeciesWS (25) to elucidate the interspecific relationships of the strain.

### Genomic DNA preparation, genome sequencing, and assembly

To isolate genomic DNA, the strain was inoculated onto 50 mL liquid medium and cultivated overnight at 30°C in a shaker at 150 rpm. The overnight culture was used in the extraction of genomic DNA by a Rapid Bacterial Genomic DNA Isolation Kit (Sangon Biotech, Shanghai, China).

Whole genome sequencing was performed using the PacBio RS and Illumina sequencing platforms. Illumina PE and PacBio (8–10 kb) libraries were constructed, and 52-Mb continuous long read (CLR) PacBio sequences were sequenced on a PacBio RS platform using a single-molecule real-time cell with an N50 sequence length of 12,035 base pairs (bp), with 47.2 Mb of the sequences being longer than or equal to 5 kb. A 400-bp Illumina sequencing library was constructed, and 2.9-Gb paired-end sequences were sequenced using the Illumina Hiseq 2000 platform. Illumina data were used to evaluate the complexity of the genome. These data were assembled using Velvet assembler version 1.2.10 with a k-mer length of 99 (29).

The complete genome sequence was assembled using both the PacBio and Illumina reads. The assembly was initially produced using an in-house assembly solution, in which *de Bruijn*-based assembly and CLR correction algorithms were integrated. The final circular step was completed manually. The genome circle was prepared using Circos version 0.64 software (http://circos.ca) (15).

### Genome annotation

Prodigal software (13) was used to predict bacterial genes. The protein sequences of the predicted genes were compared to the protein database of Clusters of Orthologous Groups (COGs) (http://www.ncbi.nlm.nih.gov/COG) (26) and the corresponding COG annotation results were obtained. BLAST (blastx/blastp 2.2.24+) was used to compare the predicted genes with the Kyoto Encyclopedia of Genes and Genomes (KEGG) database (http://www.genome.jp/kegg/genes.html), from which specific pathways involved in the corresponding genes were identified. BLAST results were analyzed by Gene Ontology annotation (http://www.geneontology.org) with the software Blast2go.

### *B. pumilus* ZB201701 and resistance to abiotic stress

Luria-Bertani (LB) agar plates with NaCl (0, 1, 3, and 5 mol L^−1^) or D-sorbitol (0, 1, 2, and 3 mol L^−1^) were used to test stress responses. In the salt and drought challenge, cultures were incubated at 30°C to a density of approximately 5×10^7^ cells mL^−1^. At this point, cell viability was assessed by plating appropriate dilutions onto agar with NaCl to a final concentration of 1, 3, or 5 mol L^−1^ or D-sorbitol to a final concentration of 1, 2, or 3 mol L^−1^ and incubating at 30°C for at least 12 h. Cell viability was calculated as follows: No. of colonies of the stress group/No. of colonies of the CK group×100. CK, 0 mol/L NaCl or D-sorbitol.

Seeds of the maize inbred line B73 were surface-sterilized, germinated, and grown in pots (length×width×height=7×7×6.6 cm) filled with soil and vermiculite (1:1 [v/v]), with four seeds per pot. Pots were placed in a greenhouse under long-day conditions (30°C, 16 h of light; 22°C, 8 h of darkness; and 65% humidity) and watered once every 3 d. At the three-leaf stage, plants were divided into the control group and treatment (*B. pumilus* ZB201701) group, with three plants in each group. In the control group, 100 mL of 0.1 mol L^−1^ NaCl was evenly applied to the soil. In the treatment group, the strain was cultured in LB medium at 30°C for 36 h and centrifugated at 200×g at room temperature for 10–20 min. Pelleted cells were resuspended in 0.1 mol L^−1^ NaCl and adjusted by further dilutions to achieve OD_600_ 0.1. One hundred milliliters of this solution (0.1 mol L^−1^ NaCl with OD_600_ 0.1 *B. pumilus* ZB201701) was then evenly applied to soil. All sampled tissues were washed by sterile distilled water and 75% ethanol. The leaf tissues of four seedlings (300 mg) were sampled after 0, 5, 10, 15, and 20 d. Maize seedling heights were measured every five d after the salt-stress treatment. Superoxide dismutase (SOD), catalase (CAT), and ascorbate peroxidase (APX) activities were measured in all samples according to previously described methods (2, 6, 23) (Table S1).

### Statistical analyses

The effects of *B. pumilus* ZB201701 on biochemical indexes under salt and simulated drought stress conditions were evaluated. The data obtained were subjected to an analysis of variance using the general linear model software Agres and Agdata, and means were compared using the least significant difference test at a probability level≤0.05.

### Nucleotide sequence accession number

The complete genome sequence of *B. pumilus* ZB201701 has been deposited in the DDBJ/ENA/GenBank databases under accession no. CP029464.

## Results and Discussion

### Organism information and classification

*B. pumilus* ZB201701 was found to be Gram-positive (Fig. S1), and shared 99.93% identity with *B. pumilus* SH-B9 and *B. zhangzhouensis* DW5-4^T^, 99.86% identity with *B. safensis* KCTC 12796B and *B. australimaris* NH7I_1^T^, and 99.58% identity with *B. altitudinis* YNP4-TSU (Table 1). Since analyses based on the 16S rRNA sequence alone are insufficient to classify species of *Bacillus*, the identities of the housekeeping genes *recA* and *atpD* in this and other strains were investigated; five strains exhibited greater than 90% identity (Table 1). The whole genome sequences of the 12 most similar strains were then analyzed via ANI using JSpeciesWS (25). The highest ANI value was 95.28% for *B. pumilus* SH-B9, which exceeded the species cut-off threshold of 95% (8). Therefore, the whole genome sequences of the two strains were compared using the online software Last (http://lastweb.cbrc.jp/) (20), which identified many differences between the two strains (Fig. S2). A comparison of the available genomes of type strains indicated that our strain is a subspecies of *B. pumilus*.

### Genome sequencing results

**Fig. 1:**
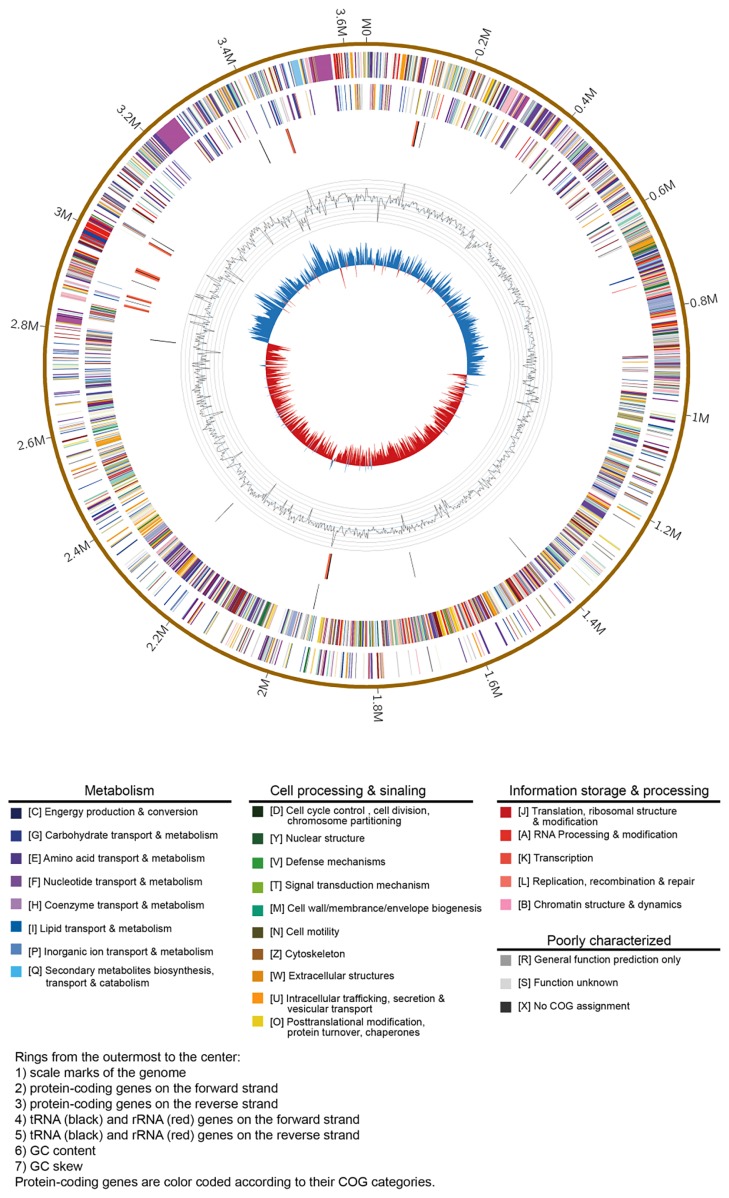
The circular chromosome of *Bacillus pumilus* ZB201701. Whole genome sequencing was performed using the PacBio RS and Illumina sequencing platforms. These sequencing data were assembled using Velvet assembler version 1.2.10 with a k-mer length of 99.

To obtain a genome sequence with no gap, a combination of the PacBio RS and Illumina sequencing platforms was used. Eighty-one contigs with lengths greater than or equal to 200 bp were included in the assembled results, with an N50 length of 161,386 bp. These results indicated that the genome of *B. pumilus* ZB201701 consisted of one circular chromosome of 3,640,542 bp (Fig. 1) and one plasmid sequence (Fig. S3) with no gaps. The G+C content of the genome was 41.86%. A total of 3,712 predicted genes were detected, 3,608 (97.2%) of which were putative protein-coding genes; 86.21% were assigned a putative function (Table 2).

**Fig. 2:**
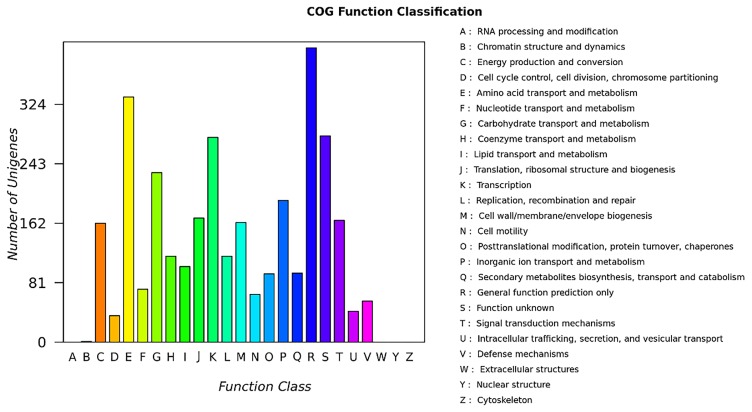
COG functional classifications of *Bacillus pumilus* ZB201701 coding sequences. Colors: blue, IslandPath-DIMOB; orange, SIGI-HMM; and green, IslandPick. Specific predictors were selected to view the results for a single method, and the manipulation settings are shown using the IslandPick display.

Based on COGs (26), the identified proteins were classified into 25 functional categories (Fig. 2). Some of the proteins were found to be involved in the response to salt and drought stress, including the amino acid transport and metabolism category (E), which contained the osmo-regulated proline transporter (27); the signal transduction mechanisms category (T) that contained the transcriptional regulatory protein DegU and signal transduction histidine-protein kinase/phosphatase DegS (16); and the transcription category (K) that contained the cold-shock protein CspB, which has been shown to improve grain yield in maize under water-limited conditions (4). A previous study indicated that the accumulation of stress-induced reactive oxygen species is counteracted by enzymatic antioxidant systems that include a number of scavengers, including SOD, CAT, and APX (21). COG functional analyses indicated several related genes, including some in the inorganic ion transport and metabolism category (P), which contains three SOD- and two CAT-related genes; some in the carbohydrate transport and metabolism category (G); and some in the function unknown category (S), which contained one APX-related gene.

### Activity of the strain under salt and simulated drought stress

**Fig. 3:**
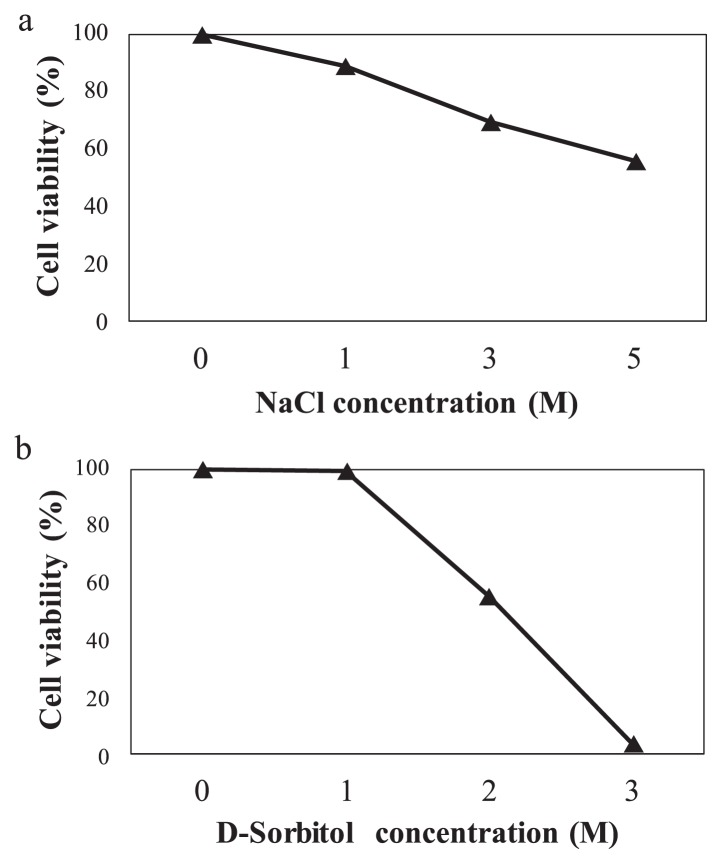
Identification of the activity of *Bacillus pumilus* ZB201701 under salt (a) and simulated drought stress (b).

*B. pumilus* strains are resistant to environmental biotic and abiotic stress (7). A strain of *B. pumilus* isolated from the penaeid shrimp has been shown to exhibit high salt tolerance (12), and *B. pumilus* ES4 from arid land soils exhibits strong plant growth-promoting activity (11). We also identified several environmental biotic and abiotic stress-related genes based on COG analyses (Fig. 2). To identify the ability of *B. pumilus* ZB201701 to resist conditions of high salt and drought, we cultured the strain on medium with different concentrations of salt and D-sorbitol. The results obtained indicated that the strain has the ability to tolerate up to 3 M D-sorbitol (approximately 55% [w/v]) and 5 M NaCl (approximately 29% [w/v]), with Cell viability of 3.5 and 55.8%, respectively (Fig. 3a and b). These results indicated that *B. pumilus* ZB201701 has the ability to resist high salt and simulated drought.

### Ability to promote the salt resistance of maize

**Fig. 4:**
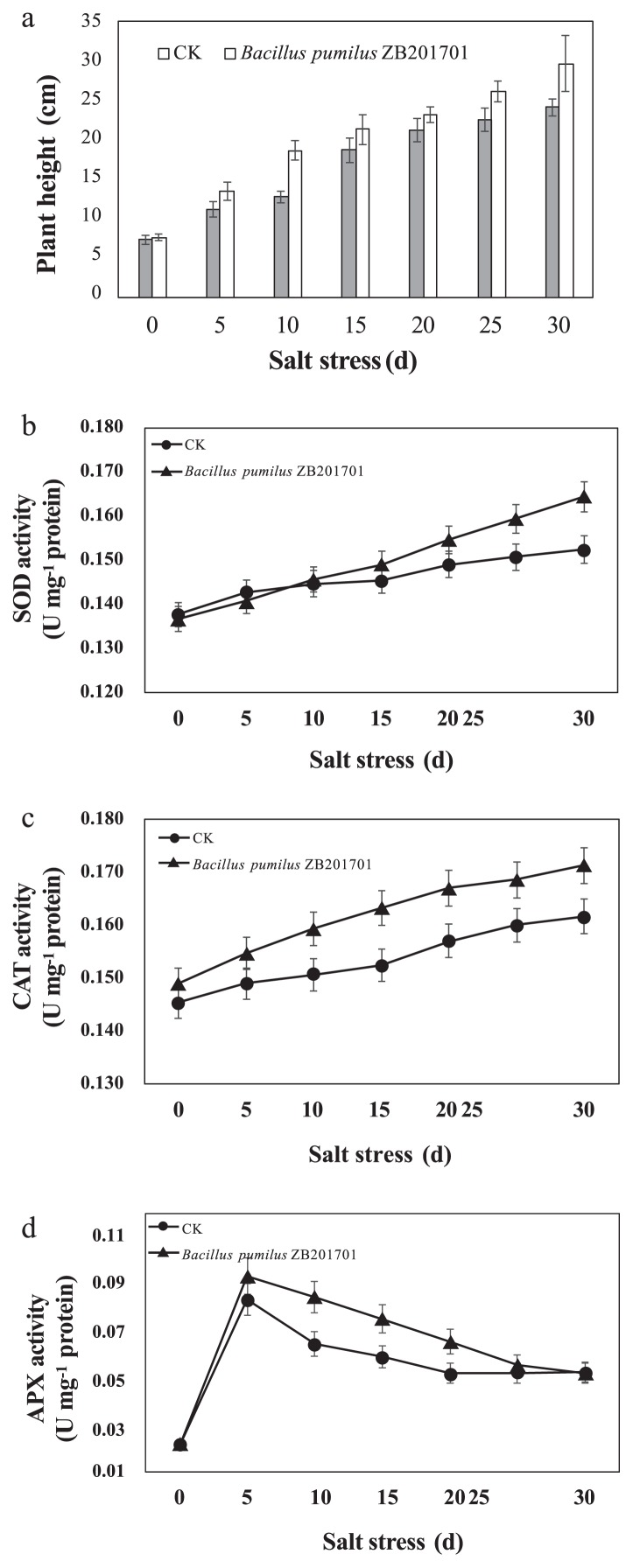
Measurement of plant height (a) and SOD (b), CAT (c) and APX (d) enzyme activities in maize under salt stress. CK: the control group, 100 mL of 0.1 mol L^−1^ NaCl was evenly applied to soil; *Bacillus pumilus* ZB201701: 100 mL solution (0.1 mol L^−1^ NaCl with OD_600_ 0.1 *B. pumilus* ZB201701) was evenly applied to soil.

The heights of maize in the *B. pumilus* ZB201701 groups were significantly greater (*P*<0.05) than those in the control group from days 5 to 30 (Fig. 4a). The results obtained also indicated that from days 25 to 30, SOD activity was significantly higher in the *B. pumilus* ZB201701 group than in the control group (*P*<0.05) (Fig. 4b). CAT activity was also markedly higher in the *B. pumilus* ZB201701 group than in the control group from days 0 to 30 (*P*<0.05) (Fig. 4c). APX activity in the *B. pumilus* ZB201701 group was higher than that in the control group on days 5, 15, 20, 25, and 30 (*P*<0.05) (Fig. 4d). These results indicate that *B. pumilus* ZB201701 promotes maize salt resistance by increasing the activities of SOD, CAT, and APX.

## SUPPLEMENTARY MATERIAL



## Figures and Tables

**Table 1:** Sequence similarity (%) with *Bacillus pumilus* ZB201701.

Strains	16S rRNA	*recA*	*atpD*	MLSA	ANI
*B. pumilus* SH-B9	99.93	97	98	ND	95.28
*B. zhangzhouensis* DW5-4^T^	99.93	94	97	ND	91.46
*B. safensis* KCTC 12796B	99.86	92	98	ND	92.40
*B. australimaris* NH7I_1^T^	99.86	92	98	ND	91.99
*B. altitudinis* YNP4-TSU	99.58	91	97	ND	89.29
*B. atrophaeus* BSS	97.32	80	82	ND	85.33
*B. subtilis* 168	97.03	81	82	ND	84.99
*B. nakamurai* NRRL B-41091^T^	97.03	81	81	ND	84.73
*B. amyloliquefaciens* DSM7^T^	96.82	80	81	ND	85.70
*B. swezeyi* NRRL B-41282	96.40	80	81	ND	84.53
*B. haynesii* NRRL B-41327^T^	96.18	80	80	ND	84.67
*B. gobiensis* FJAT-4402^T^	95.41	76	78	ND	85.28

**Table 2:** Genome features of *Bacillus pumilus* ZB201701.

Attribute	Value	% of total
Genome size (bp)	3,640,524	100
DNA coding (bp)	3,195,920	87.8
DNA G+C (bp)	1,523,704	41.86
Total genes	3,712	100
Protein coding genes	3,608	97.2
rRNA genes	24	0.65
tRNA genes	80	2.16
Genes with function prediction	3,200	86.21

## References

[1] Basu S., Rabara R., Negi S. (2017). Towards a better greener future—an alternative strategy using biofertilizers. I: Plant growth promoting bacteria. Plant Gene.

[2] Beers R.E., Sizer I.W. (1952). A spectrophotometric method for measuring the breakdown of hydrogen peroxide by catalase. J Biol Chem.

[3] Branquinho R., Meirinhos-Soares L., Carriço J.A., Pintado M., Peixe L.V. (2014). Phylogenetic and clonality analysis of *Bacillus pumilus* isolates uncovered a highly heterogeneous population of different closely related species and clones. FEMS Microbiol Ecol.

[4] Castiglioni P., Warner D., Bensen R.J. (2008). Bacterial RNA chaperones confer abiotic stress tolerance in plants and improved grain yield in maize under water-limited conditions. Plant Physiol.

[5] Forni C., Duca D., Glick B.R. (2017). Mechanisms of plant response to salt and drought stress and their alteration by rhizobacteria. Plant Soil.

[6] Giannopolitis C.N., Ries S.K. (1977). Superoxide Dismutases I. Occurrence in Higher Plants. Plant Physiol.

[7] Gioia J., Yerrapragada S., Qin X. (2007). Paradoxical DNA repair and peroxide resistance gene conservation in *Bacillus pumilus* SAFR-032. PLoS One.

[8] Goris J., Konstantinidis K.T., Klappenbach J.A., Coenye T., Vandamme P., Tiedje J.M. (2007). DNA-DNA hybridization values and their relationship to whole-genome sequence similarities. Int J Syst Evol Microbiol.

[9] Gutiérrez-Mañero F.J., Ramos-Solano B., Probanza A., Mehouachi J., Tadeo F.R., Talon M. (2001). The plant-growth promoting rhizobacteria *Bacillus pumilus* and *Bacillus licheniformis* produce high amounts of physiologically active gibberellins. Physiol. Plantarum.

[10] Han Q.Q., Lu X.P., Bai J.P. (2014). Beneficial soil bacterium *Bacillus subtilis* (GB03) augments salt tolerance of white clover. Front Plant Sci.

[11] Hernandez J.P., de-Bashan L.E., Rodriguez D.J., Rodriguez Y., Bashan Y. (2009). Growth promotion of the freshwater microalga Chlorella vulgaris by the nitrogen-fixing, plant growth-promoting bacterium *Bacillus pumilus* from arid zone soils. Eur J Soil Biol.

[12] Hill J.E., Baiano J.C.F., Barnes A.C. (2009). Isolation of a novel strain of *Bacillus pumilus* from penaeid shrimp that is inhibitory against marine pathogens. J Fish Dis.

[13] Hyatt D., Chen G.L., Locascio P.F., Land M.L., Larimer F.W., Hauser L.J. (2010). Prodigal: prokaryotic gene recognition and translation initiation site identification. BMC Bioinformatics.

[14] Kaushal M., Wani S.P. (2016). Rhizobacterial-plant interactions: strategies ensuring plant growth promotion under drought and salinity stress. Agr Ecosyst Environ.

[15] Krzywinski M., Schein J., Birol I., Connors J., Gascoyne R., Horsman D., Jones S.J., Marra M.A. (2009). Circos: an information aesthetic for comparative genomics. Genome Res.

[16] Kunst F., Rapoport G. (1995). Salt stress is an environmental signal affecting degradative enzyme synthesis in *Bacillus subtilis*. J Bacteriol.

[17] Liu Y., Lai Q., Dong C., Sun F., Wang L., Li G., Shao Z. (2013). Phylogenetic diversity of the *Bacillus pumilus* group and the marine ecotype revealed by multilocus sequence analysis. PLoS One.

[18] Lu S., Quan W., Wang S.M., Liu H.L., Tan Y., Zeng G.P., Zhang X. (2013). Correlation of soil microbes and soil micro-environment under long-term safflower (*Carthamus tinctorius* L.) plantation in China. J Environ Biol.

[19] Lugtenberg B., Kamilova F. (2009). Plant-growth-promoting rhizobacteria. Annu Rev Microbiol.

[20] Martin C.F. (2011). A new repeat-masking method enables specific detection of homologous sequences. Nucleic Acids Res.

[21] Mittler R., Vanderauwera S., Gollery M., Van Breusegem F. (2004). Reactive oxygen gene network of plants. Trends Plant Sci.

[22] Morari F., Meggio F., Lunardon A., Scudiero E., Forestan C., Farinati S., Varotto S. (2015). Time course of biochemical, physiological, and molecular responses to field-mimicked conditions of drought, salinity, and recovery in two maize lines. Front Plant Sci.

[23] Nakano Y., Asada K. (1981). Hydrogen peroxide is scavenged by ascorbate-specific peroxidase in spinach chloroplasts. Plant Cell Physiol.

[24] Numan M., Bashir S., Khan Y., Mumtaz R., Shinwari Z.K., Khan A.L., Khan A., AL-Harrasi A. (2018). Plant growth promoting bacteria as an alternative strategy for salt tolerance in plants: A review. Microbiol Res.

[25] Richter M., Rosselló-Móra R., Glöckner F.O., Peplies J. (2016). JSpeciesWS: a web server for prokaryotic species circumscription based on pairwise genome comparison. Bioinformatics.

[26] Tatusov R.L., Fedorova N.D., Jackson J.D. (2003). The COG database: an updated version includes eukaryotes. BMC Bioinformatics.

[27] Whatmore A.M., Chudek J.A., Reed R.H. (1990). The effects of osmotic upshock on the intracellular solute pools of *Bacillus subtilis*. J Gen Microbiol.

[28] Yoon S.-H., Ha S.-M., Kwon S., Lim J., Kim Y., Seo H., Chun J. (2017). Introducing EzBioCloud: a taxonomically united database of 16S rRNA gene sequences and whole-genome assemblies. Int J Syst Evol Microbiol.

[29] Zerbino D.R., Birney E. (2008). Velvet: algorithms for de novo short read assembly using de Bruijn graphs. Genome Res.

