# Supplementary material for: Analyses of the Complete Genome Sequence of the Strain Bacillus pumilus ZB201701 Isolated from Rhizosphere Soil of Maize under Drought and Salt Stress
Source: Microbes Environ. 2019 Aug 23;34(3):310–5. doi: 10.1264/jsme2.ME18096 (PMC6759339; doi:10.1264/jsme2.ME18096)

Table S1 Determination methods for SOD, CAT and APX.

SOD	Superoxide anion free radicals can reduce nitroblue tetrazolium (NBT) into the blue compound methyl hydrazine, which can be detected by absorbance at 560 nm. SOD activity was measured according to the quantity of SOD required to elicit a 50% inhibition rate. The reaction mixture contained 2.5 mL of PBS, 0.4 mL of 130 mmol L^{-1} methionine, 0.4 mL of 750 μmol L^{-1} NBT, 0.4 mL of EDTA-Na_2, 0.4 mL of 20 μmol L^{-1} riboflavin, and 0.1 mL of enzyme extract.
CAT	CAT activity was measured based on the substrate consumption rate of H_2O_2, and the concentration of H_2O_2 was measured by ultraviolet spectrophotometry at 240 nm. The reaction mixture contained 1.5 mL of PBS, 0.2 mL of 30% H_2O_2, and 0.1 mL of enzyme extract, and the reaction was stopped with 1 mL of 8% sulfuric acid.
APX	In chloroplasts, APX catalyzes the high-efficiency removal of H_2O_2, oxidizing ascorbic acid in the presence of H_2O_2 (Anjum et al. 2016). The APX enzymatic activity measurement system included 1.5 mL of PBS (pH 7.8), 1.2 mL of 0.5 mmol L^{-1} ascorbic acid, 1.2 mL of 0.1 mmol L^{-1} H_2O_2, and 0.1 mL of enzyme extract.

Fig. S1 Gram stain result of *Bacillus pumilus* ZB201701

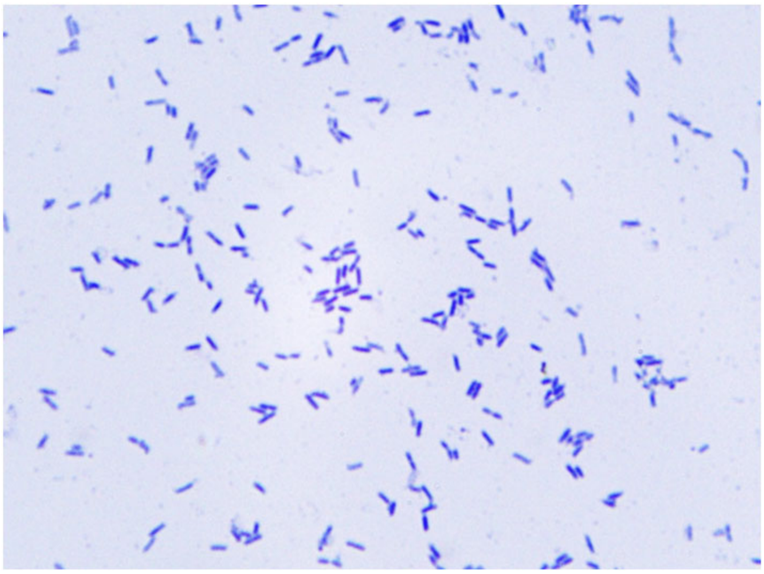

Fig. S2 Similarities of whole genome sequence from the two strains.

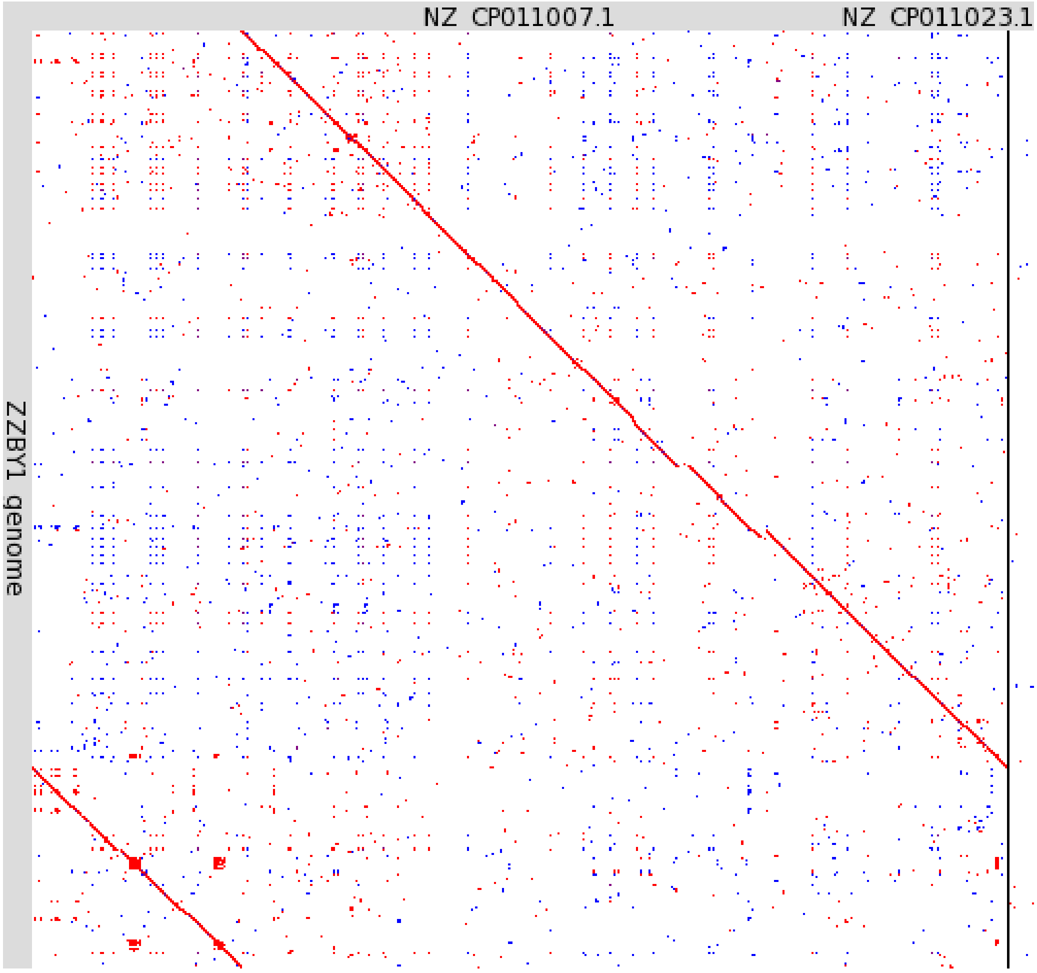

X-axis: *Bacillus pumilus* SH-B9

Y-axis: *Bacillus pumilus* ZB201701

Dot plot was performed using Last (http://lastweb.cbrc.jp/).

- probability > 0.999
- probability > 0.99
- probability > 0.95
- probability > 0.9
- probability > 0.5
- probability ≤ 0.5

Fig. S3 The circular plasmid of *Bacillus pumilus* ZB201701.

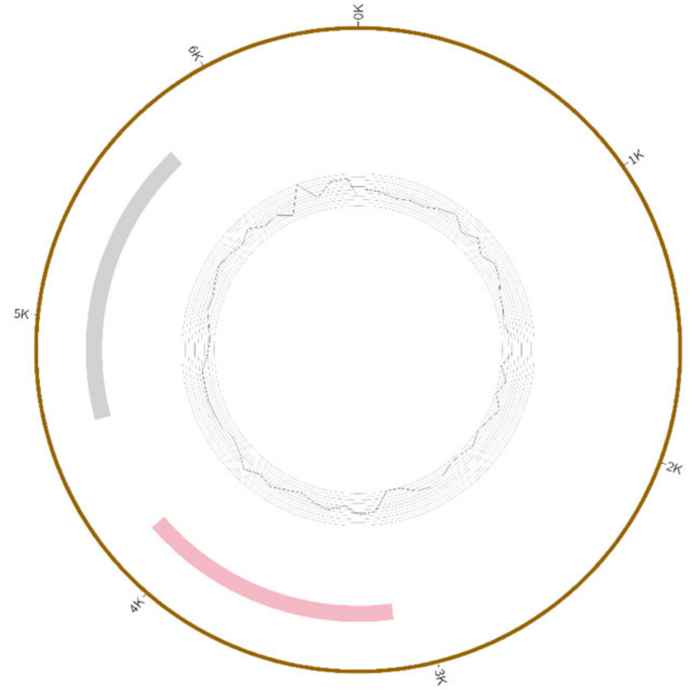

Supplement: Supplementary file 1 [file 34_310_s1.pdf]
